# A tissue‐specific screen of ceramide expression in aged mice identifies ceramide synthase‐1 and ceramide synthase‐5 as potential regulators of fiber size and strength in skeletal muscle

**DOI:** 10.1111/acel.13049

**Published:** 2019-11-06

**Authors:** Bettina Tosetti, Susanne Brodesser, Anna Brunn, Martina Deckert, Matthias Blüher, Wolfram Doehner, Stefan D. Anker, Daniela Wenzel, Bernd Fleischmann, Carola Pongratz, Franziska Peters, Olaf Utermöhlen, Martin Krönke

**Affiliations:** ^1^ Institute for Medical Microbiology, Immunology and Hygiene University Hospital Cologne Cologne Germany; ^2^ Cologne Cluster of Excellence on Cellular Stress Responses in Aging‐Associated Diseases (CECAD) Cologne Germany; ^3^ Department of Neuropathology Faculty of Medicine University of Cologne Cologne Germany; ^4^ Department of Medicine University of Leipzig Leipzig Germany; ^5^ Department of Cardiology (Campus Virchow Klinikum) German Centre for Cardiovascular Research Berlin Germany; ^6^ BIH Center for Regenerative Therapies (BCRT) Charité Universitätsmedizin Berlin Berlin Germany; ^7^ Division of Cardiology and Metabolism Department of Cardiology (Campus Virchow Klinikum) Charité Universitätsmedizin Berlin Berlin Germany; ^8^ Berlin‐Brandenburg Center for Regenerative Therapies (BCRT) Charité Universitätsmedizin Berlin Berlin Germany; ^9^ Institute of Physiology I Medical Faculty University of Bonn Bonn Germany; ^10^ Center for Molecular Medicine Cologne (CMMC) Cologne Germany; ^11^ Present address: Department of Dermatology University Hospital Cologne Cologne Germany

**Keywords:** aging, ceramide synthases, CerS1, CerS5, skeletal muscle myopathy

## Abstract

Loss of skeletal muscle mass is one of the most widespread and deleterious processes in aging humans. However, the mechanistic metabolic principles remain poorly understood. In the framework of a multi‐organ investigation of age‐associated changes of ceramide species, a unique and distinctive change pattern of C_16:0_ and C_18:0_ ceramide species was detected in aged skeletal muscle. Consistently, the expression of *CerS1* and *CerS5* mRNA, encoding the ceramide synthases (CerS) with substrate preference for C_16:0_ and C_18:0_ acyl chains, respectively, was down‐regulated in skeletal muscle of aged mice. Similarly, an age‐dependent decline of both *CerS1* and *CerS5* mRNA expression was observed in skeletal muscle biopsies of humans. Moreover, *CerS1* and *CerS5* mRNA expression was also reduced in muscle biopsies from patients in advanced stage of chronic heart failure (CHF) suffering from muscle wasting and frailty. The possible impact of CerS1 and *CerS*5 on muscle function was addressed by reversed genetic analysis using *CerS1* ^Δ/Δ^ and *CerS5* ^Δ/Δ^ knockout mice. Skeletal muscle from mice deficient of either *CerS1* or *CerS5* showed reduced caliber sizes of both slow (type 1) and fast (type 2) muscle fibers, fiber grouping, and fiber switch to type 1 fibers. Moreover, *CerS1*‐ and *CerS5*‐deficient mice exhibited reduced twitch and tetanus forces of *musculus extensor digitorum longus*. The findings of this study link CerS1 and CerS5 to histopathological changes and functional impairment of skeletal muscle in mice that might also play a functional role for the aging skeletal muscle and for age‐related muscle wasting disorders in humans.

## Graphical abstract

In skeletal muscle of aged mice and humans, the expression of *CerS1* and *CerS5* and their corresponding reaction products, C16 and C18 ceramides, are reduced. Genetic ablation of *CerS1* or *CerS5* in mice led to smaller caliber sizes of type 1 and type 2 muscle fibers and reduction of muscle strength. Patients with chronic heart failure suffering from muscle weakness presented with decreased *CerS1* and *CerS5* expression, suggesting that CerS1 and CerS5 may also be linked to functional deficiencies of skeletal muscle in humans.

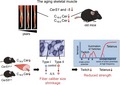

## 1 INTRODUCTION

Skeletal muscle mass and strength progressively decline during aging. The age‐related loss of muscle mass has been attributed to decrease in muscle fiber number and muscle fiber size (Nilwik et al., 2013). Aging does not affect the different muscle fiber types equally. In mammals, four major fiber types have been identified according to their respective myosin heavy chain (MyHC) composition and their metabolic profile (Schiaffino & Reggiani, 2011). Type 1 MyCH is expressed by slow‐twitch, slow‐oxidative fibers. Fast‐twitch type 2 fibers include 2A, 2X, and 2B MyCH isoforms ranging from fast‐oxidative, glycolytic to fast‐glycolytic myofibers. Whereas skeletal muscles from small mammals express all four fiber types in different proportions, humans lack fast‐glycolytic 2B fibers. Aging seems to mainly affect type 2 muscle fiber size (Lexell, 1995; Nilwik et al., 2013). Recently, transcriptomic (Chemello et al., 2011, 2019) and proteomic profiles have been described by analysis of single isolated myofibers, revealing fiber‐type‐specific changes in metabolism and sarcomere quality in aged human skeletal muscle (Murgia et al., 2017). In contrast, little is known about age‐related changes of lipids and their possible role in the aging of skeletal muscle.

Recent studies have shown that sphingolipids, including ceramide and sphingosine, accumulate in several tissues (e.g., liver and brain) during aging. Sphingolipid metabolism regulates development and lifespan in *C. elegans* (Cutler, Thompson, Camandola, Mack, & Mattson, 2014). However, the mechanisms by which ceramide synthases (CerS) may impact on aging are poorly understood.

CerS form a restricted set of C_14:0_‐C_36:0_ ceramides that serve vital functions in membrane homeostasis and cellular signaling. The mammalian CerS protein family consists of six members that differ in tissue distribution and acyl chain length usage for catalyzing the formation of dihydroceramide from dihydrosphingosine (Laviad et al., 2008; Mizutani, Kihara, & Igarashi, 2005). The distinct functions of CerS1‐6 in cell biology are beginning to be elucidated by genetic mouse models. Spontaneous recessive mutations of the *CerS1* gene cause ataxia, linked to a loss of Purkinje cells in the murine brain (Zhao et al., 2011). *CerS2*‐deficient mice develop hepatocarcinoma and myelin sheath defects (Imgrund et al., 2009; Pewzner‐Jung, Brenner, et al., 2010; Pewzner‐Jung, Park, et al., 2010). *CerS3* deficiency leads to lethal transepidermal water loss in mice (Jennemann et al., 2012) and ichthyosis in humans (Eckl et al., 2013). Using conditional *CerS4* ^Δ/Δ^ mice, we previously showed that *CerS4* is involved in the regulation of the hair follicle cycle causing age‐dependent hair loss (Peters et al., 2015). *CerS5* deficiency was reported to ameliorate high‐fat diet‐induced obesity (Gosejacob et al., 2016). *CerS6*‐deficient mice show mild behavioral abnormalities (Ebel et al., 2013). In addition, analysis of conditional *CerS6* ^Δ/Δ^ mice revealed the involvement of *CerS6* in the development of obesity in mice (Turpin et al., 2014).

Recent evidence suggests that ceramides play a role for the aging of gastric smooth muscle. The levels of *CerS2* mRNA were significantly smaller, and *CerS4‐6* levels were greater in gastric smooth muscle of aged mice associated with contractile dysfunction (Choi et al., 2015). In skeletal muscle of mice fed with high‐fat diet, CerS1‐derived C18 ceramide promoted systemic insulin resistance (Turpin‐Nolan et al., 2019), indicating an impact of ceramide in skeletal muscle metabolism.

To address possible age‐dependent and tissue‐specific roles of ceramide species and CerS isoforms, we here used an unbiased systematic approach and compared the expression of ceramide species and CerS isoforms in diverse tissues of young and aged mice. Within this framework, a consistent and distinctive pattern of decreased amounts of CerS1‐derived C_18:0_ ceramide and CerS5‐derived C_16:0_ ceramide was found in skeletal muscle of aged mice. Indeed, *CerS1* and *CerS5* are the two major *CerS* expressed in skeletal muscle (Levy & Futerman, 2010) that use C_16_ and C_18_ acyl‐CoA substrates for acylation of the free primary amine group of sphingoid bases to form the corresponding C_16:0_ and C_18:0_ ceramides. By the use of conditional *CerS1* and *CerS5* knockout mouse models, we here report on the contribution of CerS1 and *CerS*5 to skeletal muscle fiber composition and strength.

## 2 RESULTS

### 2.1 Tissue‐specific changes of ceramide and sphingomyelin species in aged mice

**Figure 1:**
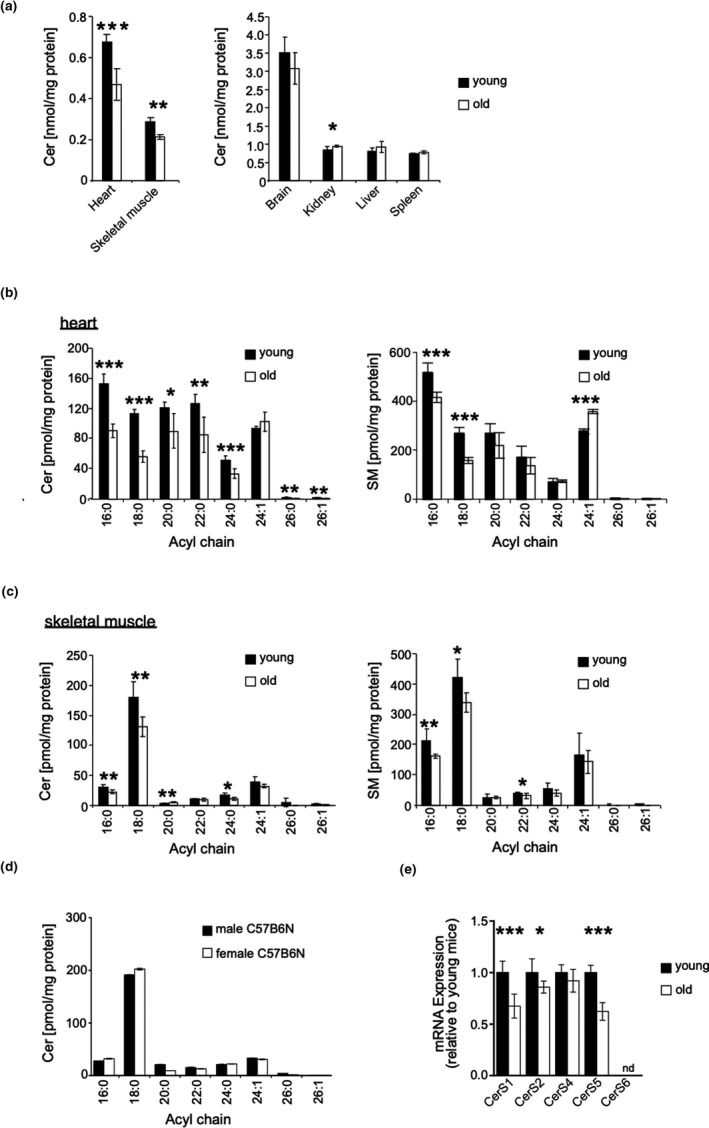
Cer C_18:0_ content and *CerS1* gene expression decrease with age in skeletal muscle. (a) Ceramide content in indicated organs and skeletal muscle from young (*n* = 3f) and old (*n* = 3f) mice quantified by LC‐ESI‐MS/MS. (b) Quantitative analysis of changes in acyl chain length distribution of Cer in heart and (c) skeletal muscle. Right panel represents acyl chain length distribution for SM in skeletal muscle. (d) Quantitative analysis of changes in acyl chain length distribution of Cer skeletal muscle of female vs. male mice (50 weeks old). (e) Analysis of *CerS* 1, 2, 4, 5, and 6 expression on mRNA level by qRT–PCR in old (*n* = 3f) vs. young mice (*n* = 3f). Values were normalized to GAPDH as internal control. Represented is mean ± *SD*. Statistical significance was assessed by two‐tailed unpaired Student's *t* test (*p < .05; **p < .01; ***p < .001)

To assess putative age‐dependent regulation of the CerS1‐6 family members by an unbiased approach, ceramide (Cer) and sphingomyelin (SM) species were analyzed by mass spectrometry in various tissues of young (6–8 weeks) and old (˃18 months) sex‐matched mice. A significant age‐dependent decrease in total Cer was observed in skeletal muscle and heart (Figure 1a). In contrast, we did not detect any significant changes of Cer in brain, kidney, liver, and spleen (Figure 1a). Cer is converted to many derivatives and thus represents only one component of a flowing equilibrium of sphingolipids including sphingomyelin, sphingosine, or glucosylceramides (GlcCer). In order to obtain a more comprehensive assessment of age‐related ceramide expression, it was therefore important to assess other sphingolipids as well. The age‐related changes of SM concentrations closely followed the changes observed with Cer. The one exception was brain, where, unlike ceramide, the SM concentration was significantly increased in aged mice (Figure S1). Analysis at the acyl chain length level revealed a generalized decrease in C_16:0_, C_18:0_, C_20:0_, C_22:0,_ and C_24:0_ Cer and C_16:0_ and C_18:0_ SM species in hearts of old mice (Figure 1b). In contrast, age‐related changes in skeletal muscle predominantly affected C_18:0_ Cer and to a lesser extent also C_16:0_, C_20:0,_ and C_24:0_ Cer (Figure 1c). Concerning SM, the levels of C_16:0_ and C_18:0_ species were diminished to comparable extent in old mice (Figure 1c). Similar profiles of sphingolipid species were observed with male and female mice (Figure 1d). A comprehensive summary of tissue‐specific distribution and age‐dependent changes of Cer, SM, and GlcCer species is depicted in Figures S3 and S4.

C18 fatty acyl‐CoA is the preferred substrate of CerS1 and C_16:0_ fatty acyl‐CoA that of CerS5 and *CerS*6, respectively. Consistently, expression of *CerS1* and *CerS5* mRNA was significantly reduced in skeletal muscle of old mice (Figure 1e). *CerS6* mRNA expression remained at the limit of detection, probably because of removal of contaminating blood cells prior to analysis (s. Material & Methods). Taken together, these observations reveal for the aged skeletal muscle a distinctive change pattern of C_16:0_ and C_18:0_ sphingolipids and their corresponding *CerS*1 and *CerS5*.

### 2.2 Age‐ and disease‐dependent changes of CerS1 and CerS5 expression in humans

**Figure 2:**
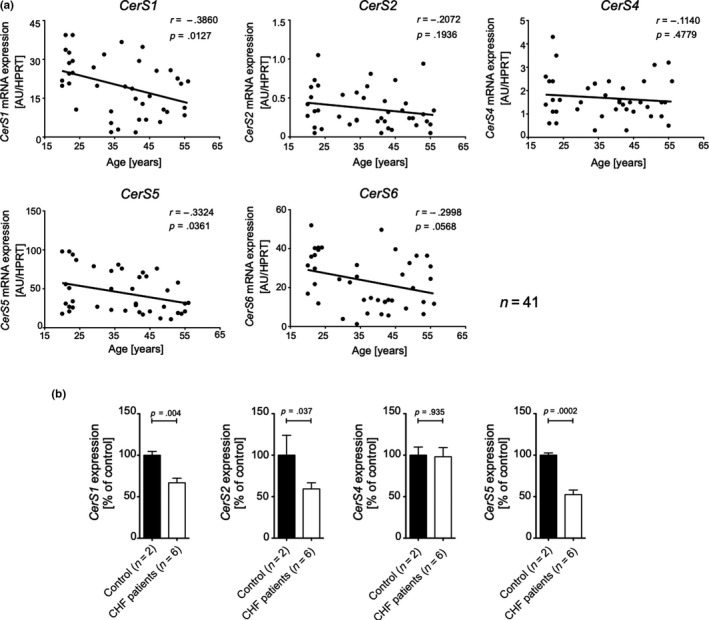
*CerS* expression declines age dependently in healthy human skeletal muscle and is further decreased in CHF patients. (a) Association between *CerS 1*, *2*, *4*, *5*, and *6* mRNA expression in skeletal muscle and age in 41 healthy humans with a wide age range (20–56 years) by Pearson's correlation (*r*). CerS mRNA levels were normalized to endogenous *HPRT* mRNA expression. (b) mRNA expression profile of indicated *CerS* in skeletal muscle biopsies from six aged patients (age: 69.1 ± 12.5 years, sex (5m/1f), BMI 24.0 ± 2.2 kg/m^2^, peak VO2 23.2 ± 4.7 ml min^−1^ mg^−1^) suffering from chronic heart failure (CHF) compared with healthy controls (1f 83y; 1m 41y), normalized to endogenous *GAPDH* mRNA expression. Shown is a representative result (*n* = 3). Shown are means ± *SD*. Statistical significance was assessed by two‐tailed unpaired Student's *t* test, and exact *p*‐values are given

We next sought to ascertain that age‐associated deregulation of *CerS1* and *CerS5* is not restricted to mice. Thus, *CerS* mRNA expression was investigated in human skeletal muscle of 41 healthy and 15 type‐2 diabetic volunteers of various ages (19–62 years, Tables S1 and S2). In human skeletal muscle, *CerS1* mRNA expression displayed the strongest inverse correlation with age (*r* = −.386, *p* < .05) (Figure 2a). A significant negative correlation coefficient of age was also evident with *CerS5* mRNA expression (*r* = −.332, *p* < .05). In contrast, the changes of *CerS2* mRNA and *CerS4* mRNA were less pronounced and did not reach statistical significance. Compared with *CerS5*, *CerS6* mRNA is expressed at very low levels in skeletal muscle and age‐related changes of expression levels did not reach statistical significance.

Previous publications implicated CerS5 and CerS6 in obesity and glucose intolerance (Gosejacob et al., 2016; Hammerschmidt et al., 2019). We, therefore, scrutinized the data for possible co‐founding effects of body mass index (BMI) and type 2 diabetes. When CerS RT–PCR values were plotted against BMI, only CerS1 mRNA expression was found to negatively correlate with BMI (*r* = −.27, *p* < .05) (Figure S5A), which was expected because, as well established, the BMI directly and strongly correlates with age (Figure S5B). The group of type 2 diabetic patients (*n* = 15, Table S2) did not show any statistically significant age‐related changes of CerS mRNA expression (Figure S6).

The mass and strength of skeletal muscle in humans are not only influenced by aging but also by sex (Doherty, 2001). We, therefore, re‐assessed the data of the cohort as depicted in Figure 2a by separately plotting male (*n* = 27) and female (*n* = 14) CerS RT–PCR values against age. Like for the entire cohort, a negative association of age with CerS1 and CerS5 mRNA expression was observed in male and female volunteers; however, none of these associations reached statistical significance (Figure S7), which is probably secondary to low number of participants.

Whether age itself or rather disease‐related muscle alterations are causally linked to *CerS1* and *CerS5* mRNA expression remained an open question. Indeed, structural and functional changes of skeletal muscle are commonly observed in patients with heart failure, leading to sarcopenia and frailty that are common complications in patients with chronic diseases (Saitoh et al., 2017; Steinbeck et al., 2015). We, therefore, analyzed the expression of *CerS* in skeletal muscle specimen from patients with chronic heart failure (CHF) (*n* = 6, Table S3). In comparison with a coeval healthy control cohort (Table S4), a reduced *CerS1* and *CerS5* mRNA expression was found in muscle biopsies from aged patients suffering from chronic heart failure (Figure 2b). *CerS2* mRNA was also down‐regulated, whereas *CerS4* mRNA expression remained unchanged, while *CerS6* expression was below the limit of detection. These few cases are consistent with the notion that CerS1 and CerS5 may also be linked to muscular disorders in chronic diseases.

### 2.3 Role of CerS1 for skeletal muscle physiology

To unravel possible age‐related phenotypic consequences caused by *CerS1* down‐regulation, conditional knockout mouse strains were generated by gene targeting using homologous recombination techniques (Figure S7A). Cre‐mediated deletion of exon 2 of *CerS1* induces a frameshift to prevent translation of the catalytical longevity assurance (LAG1) domain (D’Mello et al., 1994). The murine *CerS1* is transcribed from a monocistronic as well as a bicistronic mRNA, which also encodes growth differentiation factor 1 (*Gdf1*). *Gdf1* has been implicated in laterality/cardiac malformations (Karkera et al., 2007). As shown in Figure S7C, the deletion of exon 2 in the *CerS1* gene did not influence the transcription of *Gd*f1.

**Figure 3:**
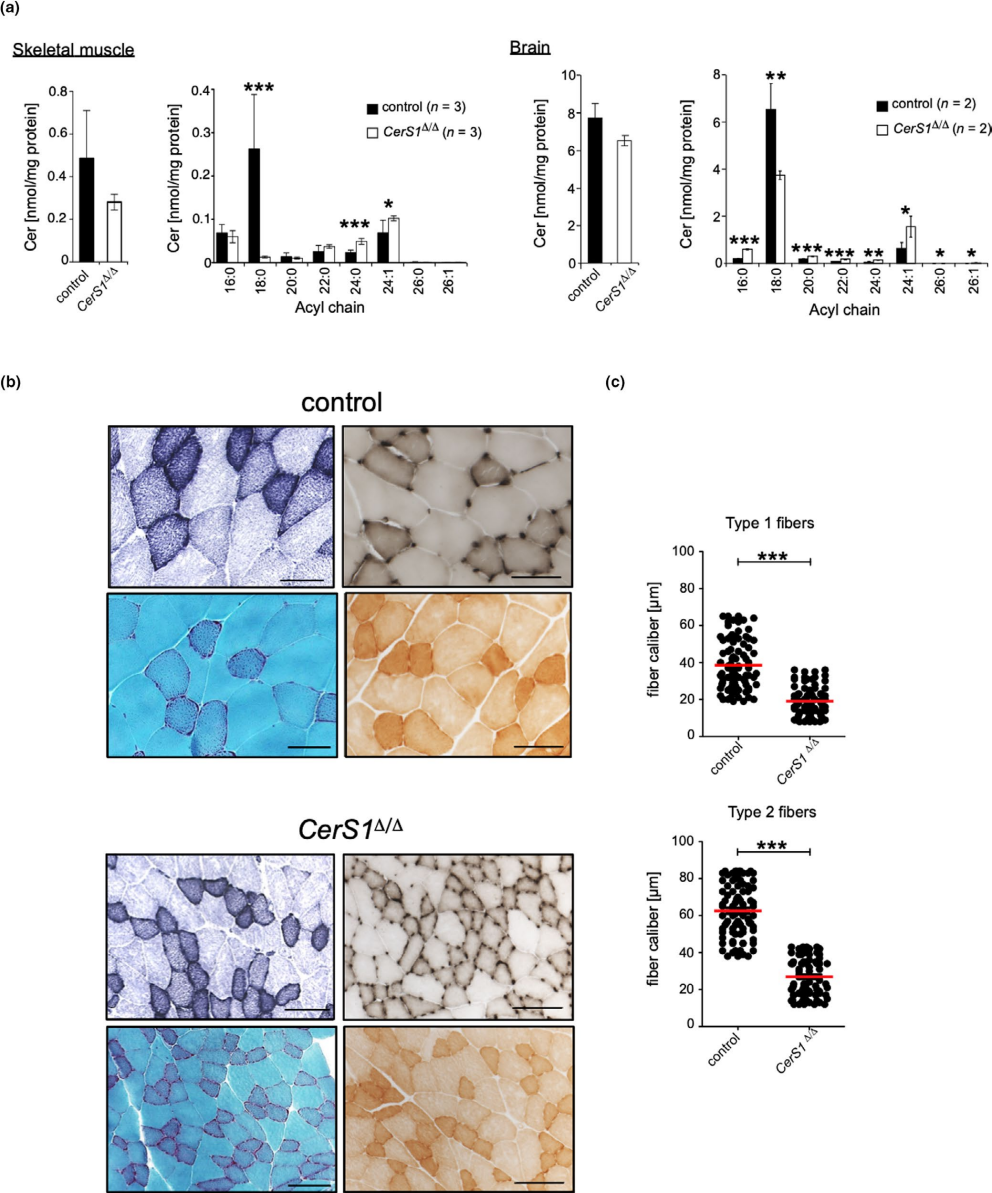
*CerS1* deficiency leads to prominent C_18:0_ Cer depletion in skeletal muscle and brain and is associated with muscle atrophy. (a) Quantitative analysis of changes in acyl chain length distribution of Cer in skeletal muscle or brain of male *CerS1* ^Δ/Δ^ mice, represented is mean ± *SD*, exact number of mice per genotype 1s indicated in figure. Each tissue sample was analyzed in duplicates. Histological analysis and determination of fiber caliber size of the *quadriceps femoris* muscle from *CerS1* ^Δ/Δ^ (*n* = 3m) and wild‐type control mice (*n* = 3m) in the age of 47 and 52 weeks old. (b) Sections were stained with red. NADH histochemistry (upper left), ATPase pH 4.4 (upper right) Gomori trichrome (lower left), or COX (lower right). Micrographs: original magnification ×200, scale bar corresponds to 100 µm. (c) Graphs depict fiber caliber size for type 1 (top) and type 2 fibers (bottom). To determine the caliber of muscle fibers (µm), serial sections with 25 high‐powered fields (HPF) of the quadriceps muscle with at least 80 muscle fibers of type 1 and type 2 fibers were counted. Each dot indicates the caliber of individual fibers, and the red bar corresponds to the mean. Statistical significance was assessed by two‐tailed unpaired Student's *t* test (*p < .05; **p < .01; ***p < .001)

As expected, deficiency of *CerS1* resulted in a marked decrease in Cer with C_18:0_ acyl chain length in skeletal muscle and brain (Figure 3a). Histological analysis of *CerS1* ^Δ/Δ^ mice was performed to identify whether the diminished levels of C_18:0_ Cer affect muscle integrity. Skeletal muscle consists of two main fiber types, one slow‐twitch, oxidative type 1 fiber and three fast‐twitch type 2 fibers, oxidative glycolytic (2A), glycolytic (2X), and fast‐glycolytic (2B) (Schiaffino & Reggiani, 2011). *Quadriceps femoris* was chosen for analysis due to the even distribution of the two main fiber types. Type 1 muscle fibers were stained with reduced NADH enzyme histochemistry and ATPase at pH4.4. As shown in Figure 3b, these two staining procedures revealed a similar proportion of type 1 and type 2 fibers. Modified Gomori trichrome histochemistry revealed some “ragged red fibers” (RRF), indicating accumulation of mitochondria (Figure 3b). In general, ≥2% of RRF in skeletal muscle is a diagnostic criterion for mitochondrial myopathy in adults (Bernier et al., 2002). We have added cytochrome *c* oxidase (COX) stainings in order to assess the nature of the RRF observed with gomori trichrome stainings. As all fibers were stained by the COX reaction, RRFs may reflect physiological decline of mitochondrial function (Larsson, 2010). The different intensities of the COX reaction correspond to type 1 (darker stains) and type 2 (lighter stains) muscle fibers, the proportion of which being consistent with those obtained with NADH and ATPase stainings.

The corresponding images of *CerS1* ^Δ/Δ^ quadriceps muscle differed in many aspects. The caliber sizes of type 1 and type 2 muscle fibers were significantly smaller (Figure 3c). Type 1 fibers showed fiber grouping, indicative for denervation. Additionally, the proportion of muscle fiber types seemed shifted to type 1 fibers. Finally, the Gomori trichrome staining showed increased numbers of RRF. Of note, RRF are not specific for an inherited mitochondrial myopathy. Indeed, RRF can develop with normal aging matching the physiological decline of mitochondrial function and accumulation of mtDNA deletions (Larsson, 2010).

**Figure 4:**
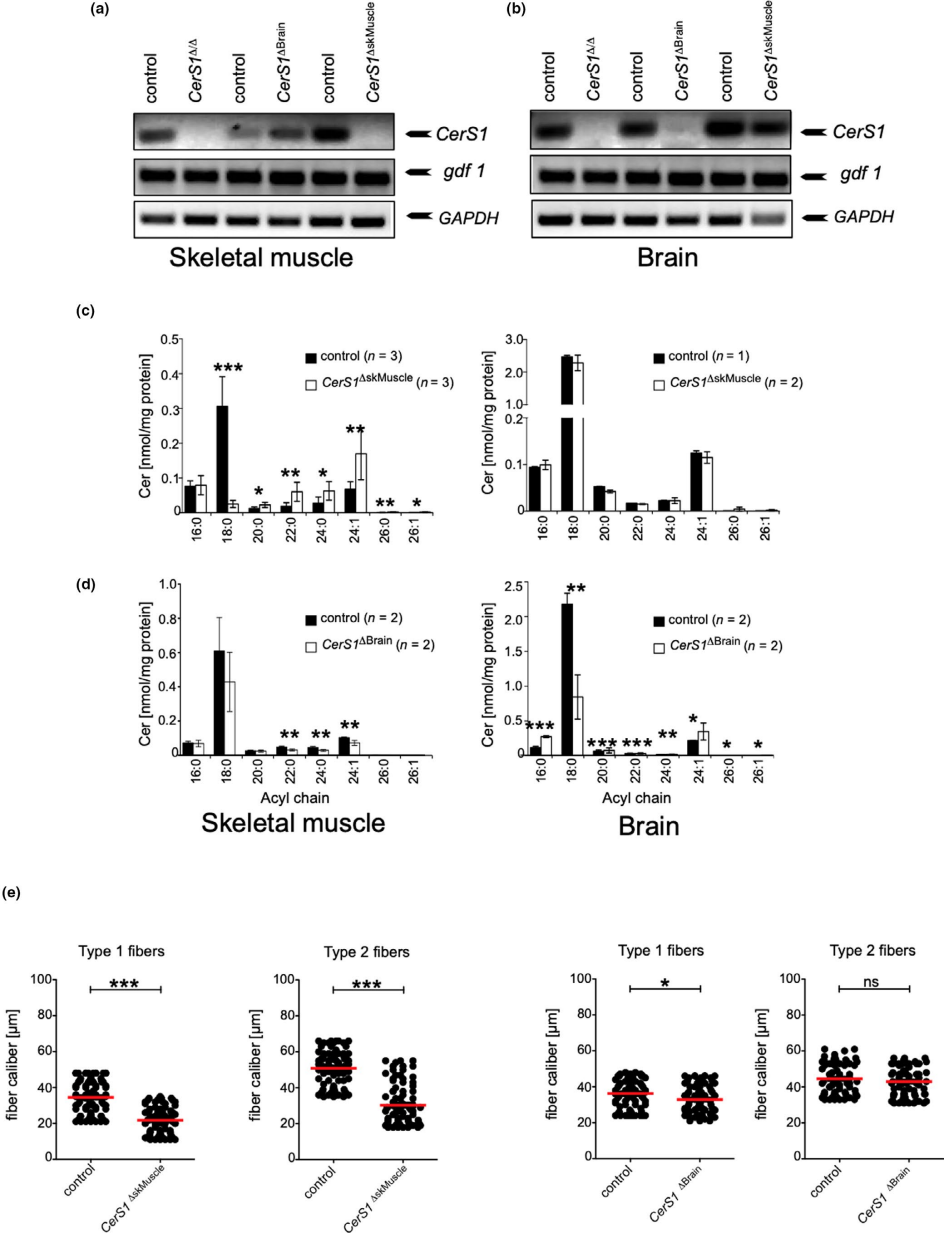
*CerS1* deficiency in skeletal muscle leads to muscle atrophy independent of purkinje cell loss. (a) RT–PCR using primers specific for *CerS1*, *gdf1,* or *GAPDH* on cDNA obtained from (a) skeletal muscle or (b) brain from indicated mice. (c) Quantitative analysis of changes in acyl chain length distribution of ceramide in skeletal muscle (left) or brain (right) of male (c) *CerS1* ^ΔskMuscle^ or (d) *CerS1* ^ΔBrain^ mice represented is mean ± *SD*, n per genotype is indicated in figure. (e) Graphs depict fiber caliber size for type 1 and type 2 fibers determined in serial sections with 25 high‐powered fields (HPF) for *CerS1* ^ΔskMuscle^ (left) or *CerS1* ^ΔBrain^ mice (right). At least 80 muscle fibers of type 1 and type 2 fibers were counted. Each dot indicates the caliber of individual fibers, and the red bar corresponds to the mean. Statistical significance was assessed by two‐tailed unpaired Student's *t* test (*p < .05; **p < .01; ***p < .001)

With regard to *CerS1* deficient mice, a well‐described cerebellar defect manifesting in ataxia (Zhao et al., 2011) may have co‐founding effects on the development of muscular atrophy as observed in *CerS1* ^Δ/Δ^ mice. To distinguish ataxia‐mediated from intrinsic muscle disorders, muscle‐ and brain‐specific deletion models for *CerS1* were generated. To this end, floxed *CerS1* mice were crossed with MCK‐Cre (Bruning et al., 1998) or Nestin‐Cre mice (Tronche et al., 1999), resulting in a muscle (*CerS1* ^ΔskMuscle^)‐ or brain (*CerS1* ^ΔBrain^)‐specific knockout of *CerS1*, respectively. Efficient deletion of *CerS1* was verified in respective tissue on cDNA level using RT–PCR (Figure 4a,b). Mass spectrometry analysis of *CerS1* ^ΔskMuscle^ mice revealed muscle‐specific down‐regulation of C_18:0_ Cer in skeletal muscle (Figure 4c, left), while as expected no changes in abundance of C_18:0_ Cer were observed in brain of these mice. Thus, *CerS1* ^ΔskMuscle^ mice indeed recapitulate the skeletal muscle‐specific partial loss of Cer C_18:0_ of aged mice and allowed us to investigate the structural and functional consequences of *CerS1* deficiency independently of the ataxia phenotype caused by full body deletion of *CerS1*. In *CerS1* ^ΔBrain^ mice, C_18:0_ Cer remained unchanged in skeletal muscle, while in brain homogenates C_18:0_ Cer was markedly reduced (Figure 3d, right). The loss of Purkinje cells is one histological hallmark of *CerS1* ^Δ/Δ^ mice (Zhao et al., 2011). As expected, like *CerS1* ^Δ/Δ^ the cerebellum of *CerS1* ^ΔBrain^ showed pronounced loss of Purkinje cells, whereas the Purkinje cells were not altered in the brains of *CerS1* ^ΔskMuscle^ mice as compared to WT mice (Figure S9A). Hence, these mice can be sensed as a model to analyze the impact of ataxia‐caused disturbed innervation on muscle morphology.

As shown in Figure 4e, the histological changes in muscles from *CerS1* ^ΔskMuscle^ mice reflected the reduction in the fiber caliber of both fiber types as observed in muscles from whole‐body *CerS1* knockout mice (Figure 4e). In contrast, the fiber caliber was not significantly altered in skeletal muscle from *CerS1* ^ΔBrain^ mice, neither in quadriceps femoris (Figure 4e right) nor in EDL (Figure S6B).

### 2.4 Role of CerS5 for skeletal muscle physiology

**Figure 5:**
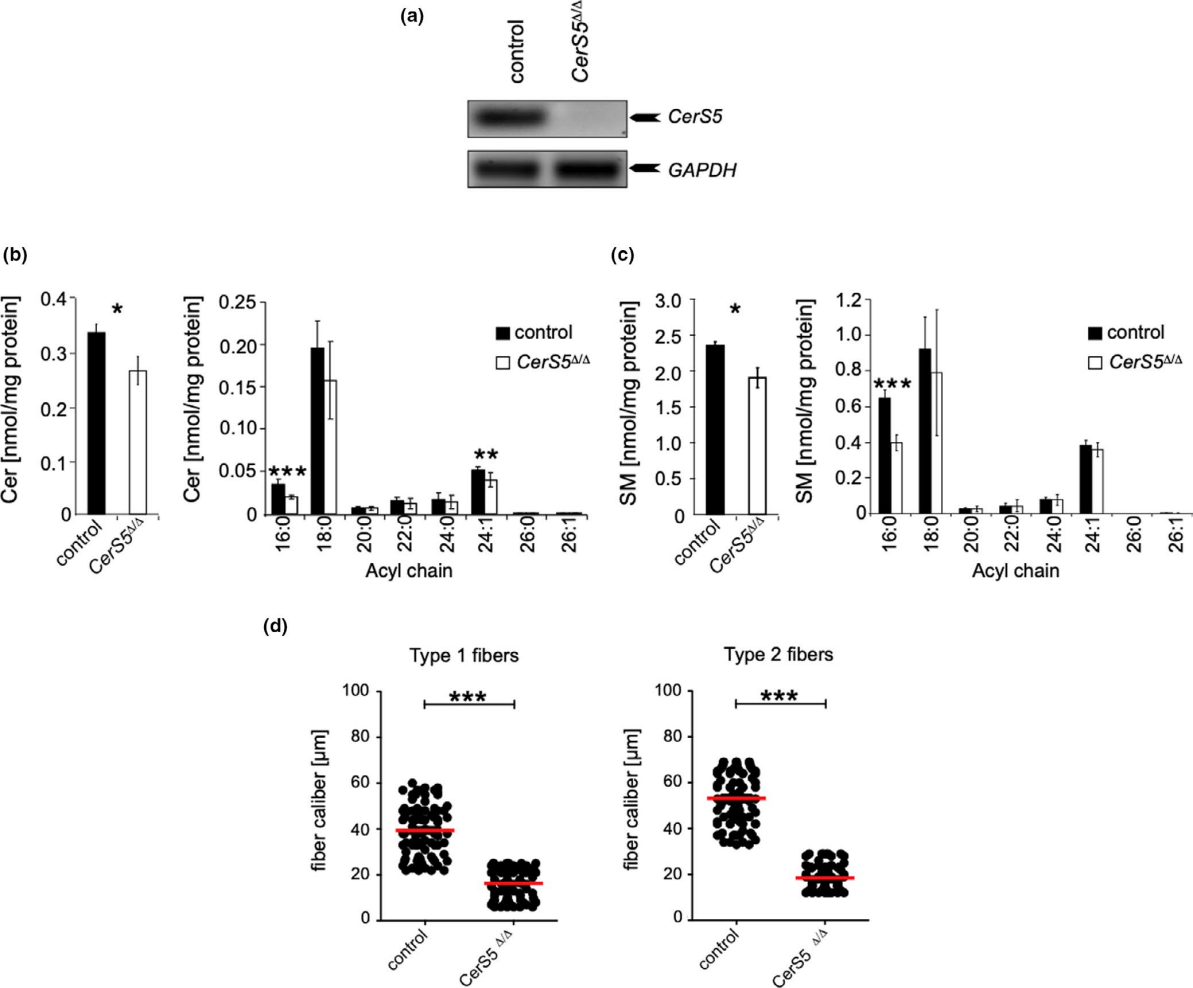
*CerS5* deficiency in skeletal muscle leads to muscle atrophy. (a) RT–PCR using primers specific for *CerS5* or *GAPDH* on skeletal muscle cDNA from *CerS5* ^Δ/Δ^ and wild‐type control mice. Quantitative analysis of changes in acyl chain length distribution of (b) Cer and (c) SM in skeletal muscle of *CerS5* ^Δ/Δ^ mice represented is mean ± *SD* from 4 male control and 5 male *CerS5* ^Δ/Δ^ mice. (d) Graphs depict fiber caliber size for type 1 and type 2 fibers. To determine the caliber of muscle fibers (µm), serial sections with 25 HPF of the quadriceps muscle with at least 80 muscle fibers of type 1 and type 2 fibers were counted. Each dot indicates the caliber of individual fibers, and the bar corresponds to the mean. Statistical significance was assessed by two‐tailed unpaired Student's *t* test (*p < .05; ***p < .001)

To address the potential role of C_16:0_ Cer for skeletal muscle homeostasis, conditional *CerS5* ^Δ/Δ^ were generated. To this end, mice carrying *loxP* sites flanking exon 4 of *CerS5* were crossed with to Cre‐deleter mice to produce *CerS5* ^Δ/Δ^ mice. The deletion of exon 4 results in a frameshift in downstream exons to prevent translation of the catalytic LAG1 domain (Figure S8B). RT–PCR analysis revealed efficient down‐regulation of *CerS5* mRNA expression (Figure 5a).

Deficiency of *CerS5* resulted in a marked decrease in Cer and SM species with C_16:0_ acyl chain lengths in skeletal muscle (Figure 5b,c). Modified Gomori trichrome enzyme histochemistry revealed ragged red fibers like in *quadriceps femoris* biopsies from *CerS1* ^Δ/Δ^ mice (not shown). Compared with *CerS1* ^ΔskMuscle^ mice, both type 1 and type 2 fibers exhibited a significantly reduced size in *CerS5* ^Δ/Δ^ mice (Figure 5d).

### 2.5 Functional consequences of CerS1 and CerS5 deficiency for skeletal muscle force

**Figure 6:**
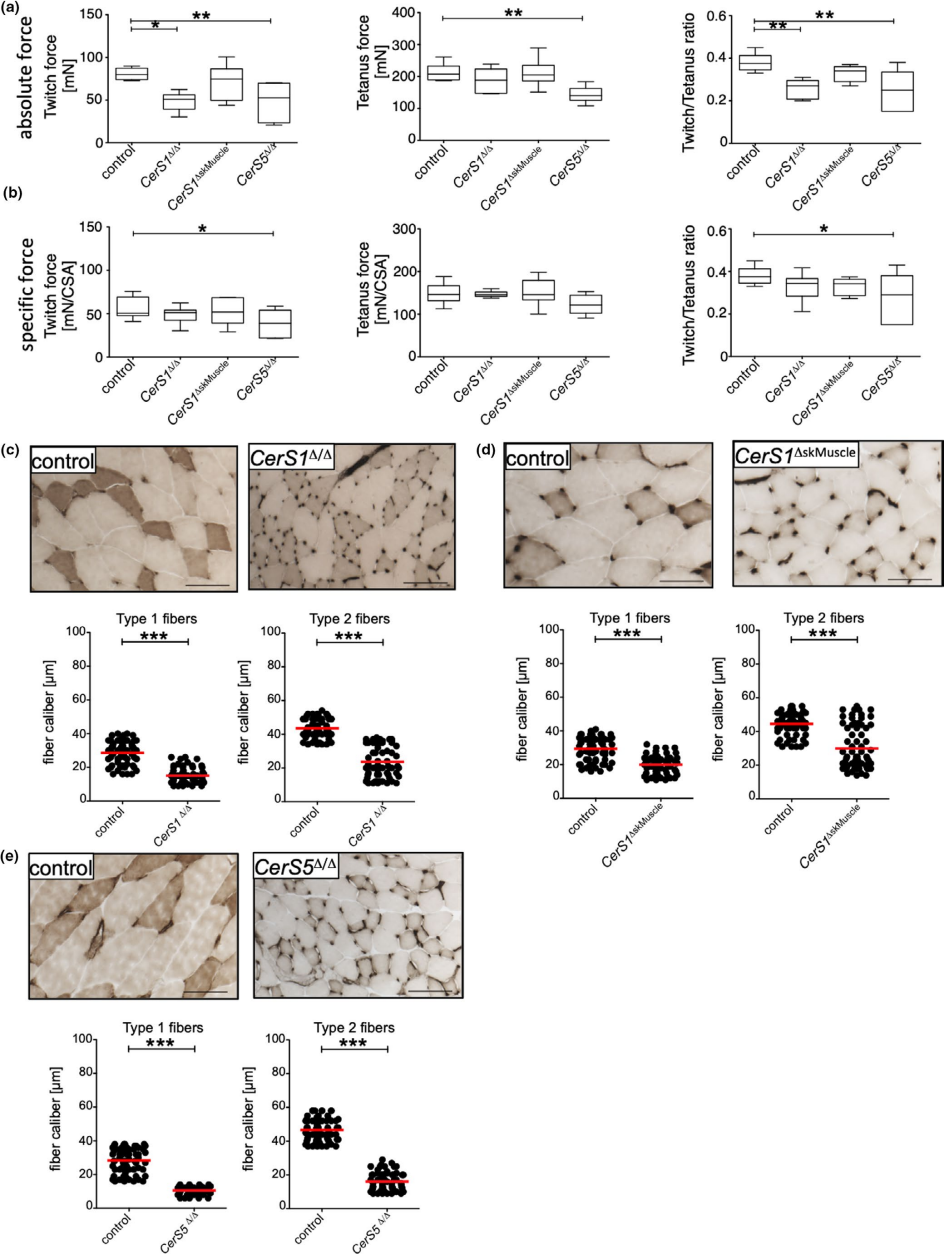
*CerS* deficiency affects EDL strength. The force upon electrical stimulation was analyzed in isolated EDL from 50 to 55 weeks old mice. (a) Absolute force values for isometric twitch (left) or tetanus stimulation (middle) or the ratio of twitch to tetanus (right) were plotted. (b) Specific force per cross‐sectional area (CSA) for isometric twitch (left) or tetanus stimulation (middle) or the ratio of twitch to tetanus (right) was plotted. Whiskers represent min and max values, and line indicates median. Statistical significance was assessed by one‐way ANOVA including Dunnett's post‐test. (c) Histological analysis and determination of fiber caliber size of the *EDL* muscle from *CerS1* ^Δ/Δ^ (*n* = 3m) *CerS1* ^Δ/Δ^ *CerS1* ^ΔSkMuscle^ and *CerS5* ^Δ/Δ^ and wild‐type control mice (*n* = 3m) in the age of 47 and 52 weeks. Sections were stained with ATPase pH 4.4. Scale bars represent 100 µm. Corresponding quantification of fiber caliber size of EDL of indicated mice and respective wild‐type control mice is depicted in graphs. Type 1 and type 2 fiber calibers were determined in 20 microscopic fields, and red line indicates mean. Statistical significance was assessed by two‐tailed unpaired Student's *t* test (*p < .05; **p < .01; ***p < .001)

Aging of skeletal muscle mostly affects fast‐twitch glycolytic type 2 fibers (Nilwik et al., 2013). We, therefore, sought to assess potential functional consequences of *CerS1* and *CerS5* deficiencies in muscle strength of the fast‐twitch *musculus extensor digitorum longus* (EDL), that consists of mainly fast‐glycolytic type 2 fibers (Schiaffino & Reggiani, 2011). In EDL from *CerS1* ^Δ/Δ^ and *CerS5* ^Δ/Δ^ mice, the twitch force, resulting from a single stimulation, was significantly reduced (Figure 6a). The tetanus force was reduced only in EDL from *CerS5* ^Δ/Δ^ mice. The twitch to tetanus ratio was significantly reduced for *CerS1* ^Δ/Δ^ and *CerS5* ^Δ/Δ^ mice, but less so in *CerS1* ^ΔskMuscle^ EDL (Figure 6a). Notably, the specific force, that is the force per cross‐sectional area (CSA), was only reduced in *CerS5* ^Δ/Δ^ mice (Figure 6b). The impairment of muscle strength correlated well with the caliber sizes of type 2 fibers in EDL from *CerS1* ^Δ/Δ^, *CerS1* ^ΔskMuscle^, and *CerS5* ^Δ/Δ^ mice, which were significantly reduced (Figure 6c–e). In contrast to the skeletal muscle‐specific, CerS1 KO mice, *CerS5* ^Δ/Δ^ mice and *CerS5* ^Δ/Δ^ mice showed fiber type grouping, indicative for denervation processes in whole‐body knockouts.

## 3 DISCUSSION

The sphingolipid composition of cellular membranes changes during aging, which is thought to be relevant for the signaling function of membrane integral receptors or for secretion of cytokines or hormones. We here systematically analyzed tissue‐specific and age‐related changes of Cer, SM, and glucosylceramides (GlcCer) species in brain, heart, liver, spleen, kidney, and skeletal muscle. The most striking observation was a consistent decline of C_16:0_ and C_18:0_ Cer and SM in skeletal muscle of aged mice and a corresponding decline of *CerS1* mRNA and *CerS5* mRNA expression. Genetic deficiencies of *CerS1* and *CerS5* showed reduced caliber sizes of type 1 and type 2 muscle fibers, ragged red fibers as histological signs of myopathy, and reduced strength. In humans, *CerS1* and *CerS5* mRNA expression declined not only age dependently but was also associated with chronic heart failure, suggesting that age‐ and critical illness‐related changes of specific Cer species contribute to myo‐pathological disorders.

Although the concentrations of individual Cer species showed significant age‐associated changes (Figure S3B), the overall concentrations of Cer remained remarkably constant in brain, kidney, liver, and spleen (Figure 1). In contrast, the overall concentrations of Cer declined in heart and skeletal muscle, suggesting decreased rates of overall Cer synthesis. It is important to emphasize that ceramides are in a flowing equilibrium with other sphingolipids; that is, steady‐state concentrations of Cer do not solely reflect the rate of Cer synthesis; and they are also secondary to Cer metabolism including hydrolysis by ceramidases, glucosylation by ceramide glucosyltransferase, or conversion to SM by sphingomyelin synthases. Notwithstanding, the overall changes of SM concentrations observed in aged mice closely recapitulated the changes observed with ceramides (Figure 1b,c and Figures S1 and S2). Thus, the decline of Cer and SM observed in heart and skeletal muscle presumably originates from a corresponding age‐associated decline of overall CerS activity. Cer and SM were the quantitatively predominant sphingolipid species, as the tissue concentrations of GlcCer were generally 10‐ to 100‐fold smaller compared with Cer and SM. Of incidental note, the prevailing GlcCer species were composed of C_18:0_ or C_24:1_ ceramides in brain and skeletal muscle and C_16:0_ and C_24:1_ Cer in all other tissues (Figure S2), consistent with a previous report on encephalopathogenic reduction of GalCer in *CerS2*‐deficient mice (Ben‐David et al., 2011).

At a higher resolution level, many age‐dependent quantitative changes of individual Cer species were detected depending on the tissue investigated. The pattern of Cer and SM species distribution was consistent with the published tissue‐specific expression levels of *CerS1‐6* mRNAs (Laviad et al., 2008; Levy & Futerman, 2010). For example, in brain, the predominant C_18:0_ Cer and C_24:1_ Cer reflect the abundant *CerS*1 and *CerS*2 expression, respectively. Here, the age‐associated loss of C_18:0_ Cer was accompanied by increases in C_16:0_ and C_24:1_ Cer concentrations to compensate for an equal overall concentration of Cer species in the brain. Similarly, in liver, kidney, and spleen, the down‐regulation of a specific Cer species was compensated by an up‐regulation of another Cer species yielding a balanced overall concentration of Cer and SM species in aged mice. This compensatory principle is consistent with a previously reported concept of a circular network of co‐regulated enzymes of lipid metabolism (Koberlin et al., 2015), where the inactivation of a particular enzyme will induce adaptive mechanisms including the compensatory up‐regulation of functionally related enzymes. One prominent example describes a compensatory up‐regulation of *CerS6*‐derived increase of C*16:0* Cer in haplodeficient *CerS2* mice, which mediated susceptibility to diet‐induced steatohepatitis and insulin resistance (Raichur et al., 2014). These compensatory lipid co‐regulatory networks make it difficult, in general, to assign a specific functional phenotype to a specific enzyme involved in lipid metabolism.

The uncompensated yet selective age‐associated loss of C_16:0_ and C_18:0_ ceramides was of special interest. The down‐regulation of *CerS* with substrate preference for C_16:0_ and C_18:0_ acyl chains, *CerS1* and *CerS5*, in aged mice pointed to a possible role of these CerS in skeletal muscle aging. As *CerS1* and *CerS5* produce the most prevailing Cer species in skeletal muscle (Figure 1c and Levy & Futerman, 2010), skeletal muscle seemed especially amenable to analysis by genetically defined mouse strains with inactivated *CerS1* and *CerS5* genes. With respect to *CerS1*, the cerebellar damage detected in *CerS1* ^Δ/Δ^ mice shows all signs of ataxia with severe movement disorders, which could have an indirect yet co‐founding effect on muscle homeostasis. We, therefore, generated a muscle‐specific *CerS1*‐deficient strain of mice (*CerS1* ^ΔskMuscle^) that did not show cerebellar Purkinje cell damage and ataxic movement disorders. Thus, *CerS1* ^ΔskMuscle^ mice allowed us to investigate the functional role of *CerS1* in skeletal muscle. Histological analysis of *CerS1* ^ΔskMuscle^ and *CerS5* ^Δ/Δ^ mice showed aberrant numbers of ragged red fibers. Slow (type 1) and fast (type 2) fibers of *quadriceps femoris* of both *CerS1* ^ΔskMuscle^ and *CerS5*‐deficient mice had significantly reduced caliber sizes. The reduced caliber sizes were most pronounced in the *extensor digitorum longus* (EDL) from *CerS5* ^Δ/Δ^ mice, which corresponded well with reduced twitch and tetanus forces. Lack of *CerS1* or *CerS5* has been associated with improvement of systemic glucose homeostasis via increase in Fgf21 from skeletal muscle (Turpin‐Nolan et al., 2019) and insulin resistance (Gosejacob et al., 2016), respectively, indicating that the negative effects of *CerS1* and *CerS5* on skeletal muscle fiber size and strength are not secondary to insulin resistance. Together, our results demonstrate an age‐associated, selective, and significant loss of C_16:0_ and C_18:0_ ceramides and a corresponding decline of *CerS1* and *CerS5* in skeletal muscle of humans and mice. Furthermore, genetic mouse models for *CerS1* and *CerS5* deficiency revealed a novel role of *CerS1* and *CerS5* for the regulation of skeletal muscle caliber fiber sizes and strength.

Notably, reduced *CerS1* and *CerS5* mRNA expression was observed in chronically ill patients suffering from chronic heart failure (Figure 2), suggesting that decline of *CerS1* and *CerS5* gene expression is functionally relevant for the development of critical illness‐associated myopathies (Latronico & Bolton, 2011; Saitoh et al., 2017). This observation is of special interest in the context of muscle wasting and sarcopenia in the elderly. Age‐related loss of skeletal muscle mass and strength is a progressive and deteriorating process during aging. The decline in muscle mass is most prominent in the lower limbs (Janssen, Heymsfield, Wang, & Ross, 2000) and is mainly attributed to reduction in fast (glycolytic) muscle fibers (type 2) (Nilwik et al., 2013). Aged muscles are characterized by defective mitochondrial biogenesis and reduced muscle mass. Using single muscle fiber proteomics, Murgia and coworkers recently reported that whereas mitochondrial content declined with aging in both slow (oxidative, type 1) and fast fiber types (type 2), glycolysis and glycogen metabolism are up‐regulated in slow, but down‐regulated in fast muscle fibers (Murgia et al., 2017). The age‐dependent deleterious structural and functional deficits in mitochondrial function and muscle mass can be exacerbated by diseases and sedentary lifestyle (Cartee, Hepple, Bamman, & Zierath, 2016). In particular, critical illness conditions including chronic heart failure seem to be the major driver of acute skeletal muscle wasting (Latronico & Bolton, 2011; Puthucheary et al., 2013; Steinbeck et al., 2015). The negative effects of *CerS1* or *CerS5* deficiencies on skeletal muscle fiber caliber size and strength in mice suggest that the declines of *CerS1* and *CerS5* expression found in both, critical illness conditions and age, may aggravate each other to produce muscle wasting phenotypes such as sarcopenia in elderly humans.

The results of this study raise many questions concerning the mechanisms of CerS1 and CerS5 action. As a metabolic organ, skeletal muscle is closely linked to glucose homeostasis. Indeed, previous publications implicated CerS1 and CerS5 in obesity and glucose intolerance (Hammerschmidt et al., 2019; Turpin et al., 2014; Turpin‐Nolan et al., 2019) (Gosejacob et al., 2016) Specifically, CerS1‐derived C18‐ceramide in skeletal muscle was shown to enhance whole‐body glucose metabolism in obesity by increasing the muscle‐derived adipokine Fgf21 (Turpin‐Nolan et al., 2019). The impact of CerS5 is controversial. Previously, a whole‐body knockout of CerS5 was reported to ameliorate high‐fat diet‐induced obesity (Gosejacob et al., 2016). Hammerschmidt and coworkers showed that CerS6‐derived C16 ceramide but not CerS5‐derived C16 ceramide protects from high‐fat diet‐induced obesity and insulin resistance. These conflicting results are probably secondary to a different CerS5 gene targeting strategy of Gosejacob et al., who used different ES cells lacking the X‐linked hypoxanthine phosphoribosyltransferase gene and replaced the entire *CerS5* gene by a bacterial LacZ gene. This extensive engineering of the *CerS5* locus may have resulted in additional regulatory effects in this study. We here report that *CerS1* mRNA expression, indeed, negatively correlated with the body mass index (BMI, *r* = −.27, *p* < .05; Figure S5A). However, since the BMI significantly increases with age (Figure S5), BMI may represent a coincidental rather than co‐founding factor for *CerS1* mRNA expression. Interestingly, the group of type 2 diabetic patients (*n* = 15) did not show any statistically significant age‐related changes of CerS mRNA expression (Figure S6), which might be secondary to the low numbers of participants. A combination of CerS gene knockout technologies and fiber‐specific transcriptome as well as proteome analysis will be most suitable to provide mechanistic insights into downstream targets of CerS1‐ and CerS5‐derived C18 and C16 ceramides, respectively, and their function in signaling pathways controlling muscle fiber‐type‐specific gene programs in the aging skeletal muscle.

## 4 EXPERIMENTAL PROCEDURES

### 4.1 Human subjects

Skeletal muscle from healthy volunteers (age 19–63 years): the study was approved by the local ethics committee (approval numbers: 159‐12‐21052012 and 017‐12‐23012012), and participants gave their informed written consent. The study with muscle biopsies from patients with chronic heart failure was approved by the ethics committee of the University of Berlin, Berlin, Germany. The study protocol was approved by the Institutional Review Board, and written informed consent was obtained from all subjects before study entry.

### 4.2 Mice

Generation of C57BL/6‐CerS1^tm1735Arte^ mice and C57BL/6‐CerS5^tm1738Arte^ mice: CerS1^Δ/Δ^ and CerS5 ^Δ/Δ^ mice were generated according to standard protocols by TaconicArtemis^©^. The schemes are depicted in Figure S8A,B, respectively. Skeletal muscle (CerS1^ΔskMuscle^)‐ or brain (CerS1^ΔBrain^)‐specific knockout mice were generated by crossing of *CerS1* ^fl/fl^ mice with MCK‐Cre (Bruning et al., 1998) or Nestin‐Cre (Tronche et al., 1999) mice, respectively. Aged mice: 18‐month‐old C57BL/6 were purchased from Charles River Laboratories, France. Mice were kept under specific pathogen‐free conditions at the animal facilities of the Medical Center, University of Cologne. Mice were allowed ad libitum access to food and water and maintained in a facility with a 12‐hr light/dark cycle at 22–24°C. Experiments were performed in accordance with the Animal Protection Law of Germany in compliance with the Ethics Committee at the University of Cologne with age‐ and sex‐matched groups of 7‐ to 12‐week‐old mice. In accordance with these rules, our protocol was approved by the North Rhine‐Westphalia State Agency for Nature, Environment and Consumer Protection. Permission to maintain and breed mice was issued by the Department for Environment and Consumer Protection–Veterinary Section, Cologne, North Rhine‐Westphalia, Germany.

### 4.3 Lipid analysis

Sphingolipid levels in mouse tissues were determined by liquid chromatography coupled to electrospray ionization tandem mass spectrometry (LC‐ESI‐MS/MS). Mouse tissue samples were homogenized in water (approximately 100 mg tissue/ml) using the Precellys^®^24 homogenizer (Peqlab). The protein content of the homogenate was routinely determined using bicinchoninic acid. 100 µl of tissue homogenate was used for lipid extraction. Lipid extraction and LC‐ESI‐MS/MS analyses were performed as previously described (Schwamb et al., 2012).

### 4.4 Gene expression analysis

Prior to organ preparation, anaesthetized mice were perfused with isotonic (0.9%) NaCl solution, to minimize contamination with blood cells. Tissues from young (6–8 weeks) and old (˃19 months) wt as well as 6‐ to 8‐week‐old CerS1^Δ/Δ^ and CerS5^Δ/Δ^ mice were immediately frozen on dry ice and stored at −80°C to prevent RNA degradation. For RNA Isolation tissue was homogenized in Trizol^®^ (Life Technologies) using the Precellys^®^24 homogenizer. Muscle biopsies from CHF patients and respective controls were homogenized like murine tissue.

Total RNA was isolated using Trizol^®^ and chloroform/isoamylalcohol (24:1). Genomic DNA was removed prior to cDNA synthesis (DNaseI; Fermentas). CDNA was synthesized using the RevertAidTM Premium First Strand cDNA Synthesis Kit (Fermentas) based on 1.5 µg of total RNA per reaction. Quantitative real‐time PCR was performed on the resulting cDNA using the LightCycler 480 SYBR Green I Master (Roche Applied Sciences) with primers for mouse or human *GAPDH*, *CerS1, CerS2, CerS4, CerS5, and CerS6*. Data analysis was performed based on linear regression of the logarithmic (log) fluorescence values per cycle using the LinRegPCR program (Ramakers, Ruijter, Deprez, & Moorman, 2003), and target gene expression was normalized to the reference gene *GAPDH*.

CerS mRNA expression in old vs. young humans was measured by quantitative real‐time RT–PCR using predesigned TaqMan assays and the Brilliant SYBR Green QPCR Core Reagent Kit from Stratagene. Fluorescence was detected on an ABI PRISM 7000 sequence detector (Applied Biosystems). Total RNA was isolated using Trizol^®^, and 1 µg RNA was reverse transcribed with standard reagents (Life Technologies). Quantification of the given *CerS* mRNA was calculated relative to *HPRT* mRNA expression. Primer sequences and catalog No. for Taqman assays are given in Table S1.

### 4.5 Histopathology

Skeletal muscles of sex‐ and age‐matched *CerS1* ^Δ/Δ^, *CerS1* ^ΔskMUSCLE^, and respective wt control mice were mounted on thick filter paper with Tissue Tek OTC compound (Miles Scientific), snap‐frozen in isopentane (Fluka), precooled on dry ice, and stored at −80°C until preparation of serial 10 µm frozen sections. Cryosections were stained with reduced NADH enzyme histochemistry, ATPase pH4.4, Gomori trichrome, or cytochrome c oxidase (COX) according to standard protocols. Muscle fiber caliber was assessed in 20 microscopic fields per muscle at a microscopic magnification of 200.

### 4.6 Muscle force measurements

After mice were sacrificed, the *extensor digitorum longus* (EDL) muscles were dissected and kept submerged under oxygenated Krebs’ preparation buffer at RT.

The muscle tendons were mounted between a lever arm and the hook of a force transducer of the myograph. Under isometric twitch stimulations, the length of the muscles was adjusted to the length that correlated with the maximum isometric twitch force. Subsequently, isometric twitch stimulations were exerted (2 V, 1 ms) and resulting forces were measured. After that, tetanic forces (400 ms, 125 Hz) were recorded. Specific force (mN/CSA) was calculated by relating the absolute force value to length and weight of the muscle using the formula: force (mN) × length (mm) × constant_murine muscle_ (1.06 mg/mm^3^) × constant_EDL_ 0.44 × mass (mg)^−1^.

### 4.7 Statistical analyses

Data are expressed as the mean ± *SD* unless stated otherwise. To analyze statistically significant differences between two groups, two‐tailed unpaired Student's *t* test was performed. For multiple comparisons ANOVA with Dunnett's post‐test were performed. *p* values <.05 were considered statistically significant and marked with *. *p* values <.01 were marked with **, and *p* values <.001 were marked with ***. Statistical analyses were performed with GraphPad Prism version 5.0 for mac.

## CONFLICT OF INTEREST

The authors declare no conflict of interest.

## AUTHOR CONTRIBUTIONS

BT planned, performed, and analyzed experiments. SB conducted lipidomic analysis. AB and MD planned and performed histopathological analyses. MB collected samples, planned, performed, and analyzed CerS expression in muscle biopsies from healthy human donors. SDA and WD collected and contributed CHF patient samples. BF and DW planned and performed muscle force measurements and analysis. FP, OU, and CP performed experiments and supported animal‐related work. MK conceived the study, evaluated experimental data, and wrote the manuscript. All authors read and approved the manuscript.
