# Supplementary figures and images for: A tissue‐specific screen of ceramide expression in aged mice identifies ceramide synthase‐1 and ceramide synthase‐5 as potential regulators of fiber size and strength in skeletal muscle
Source: Aging Cell. 2019 Nov 6;19(1):e13049. doi: 10.1111/acel.13049 (PMC6974707; doi:10.1111/acel.13049)

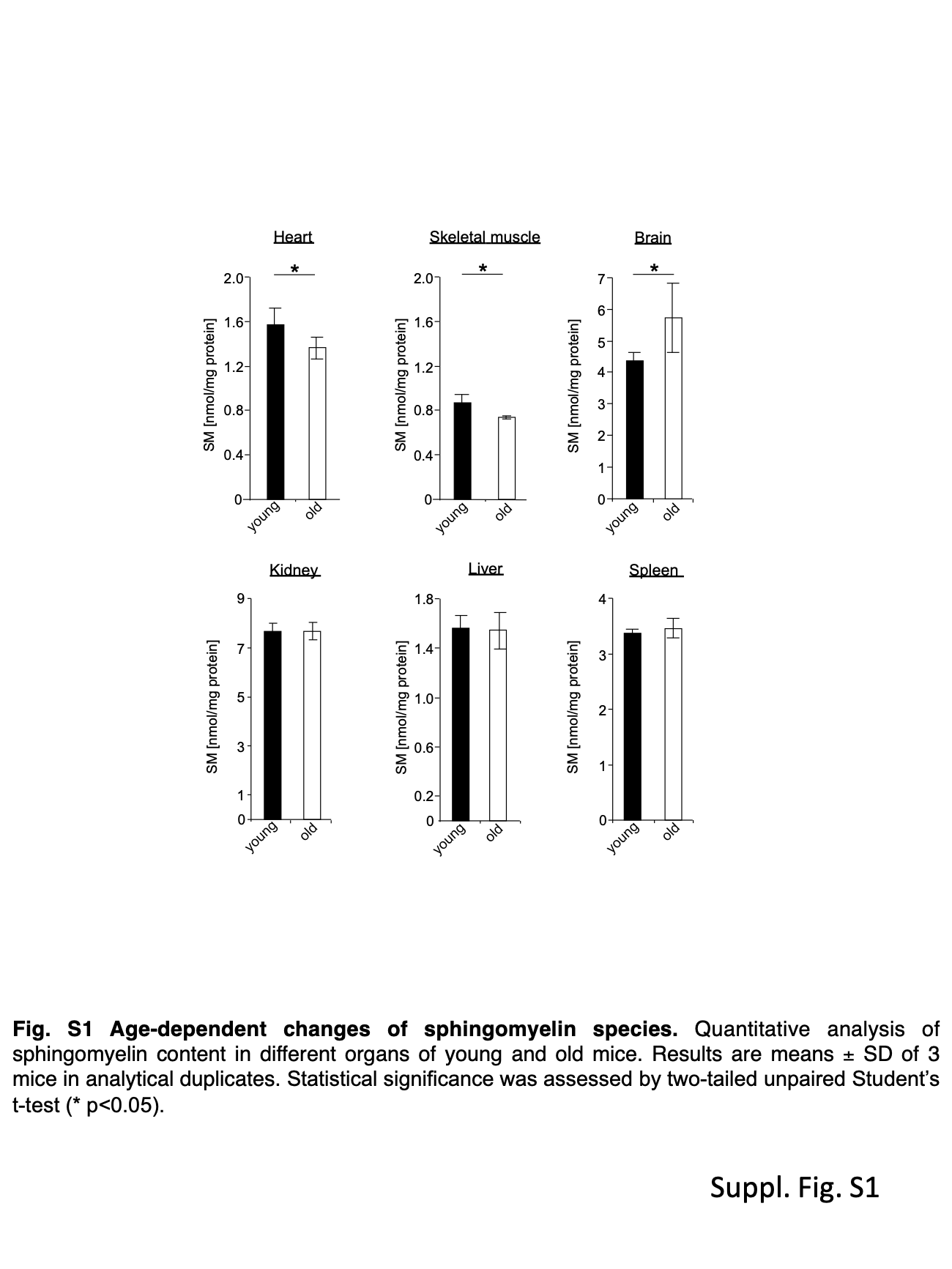

Supplement: Supplementary file 1 [file ACEL-19-e13049-s001.tiff]

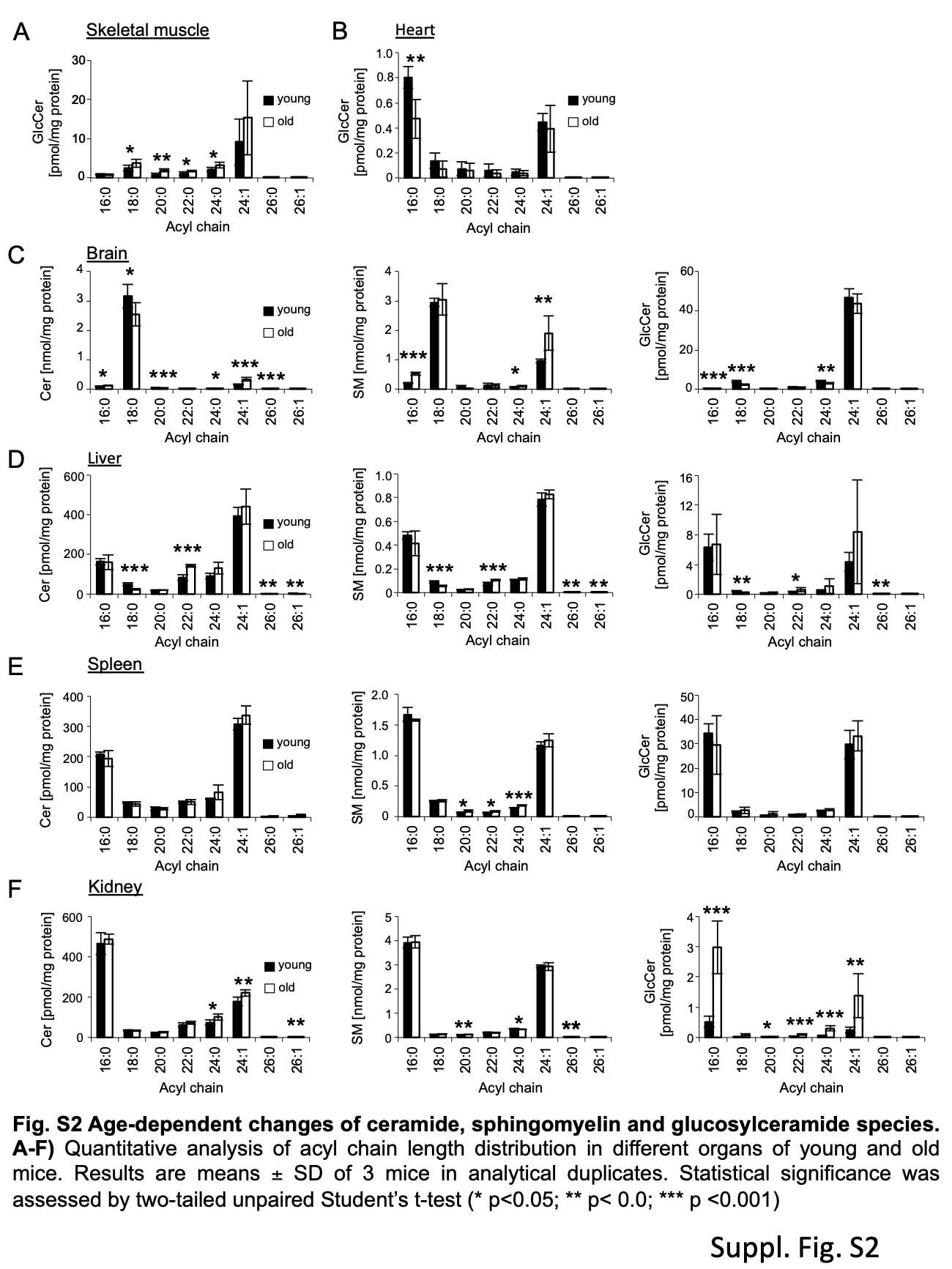

Supplement: Supplementary file 2 [file ACEL-19-e13049-s002.tiff]

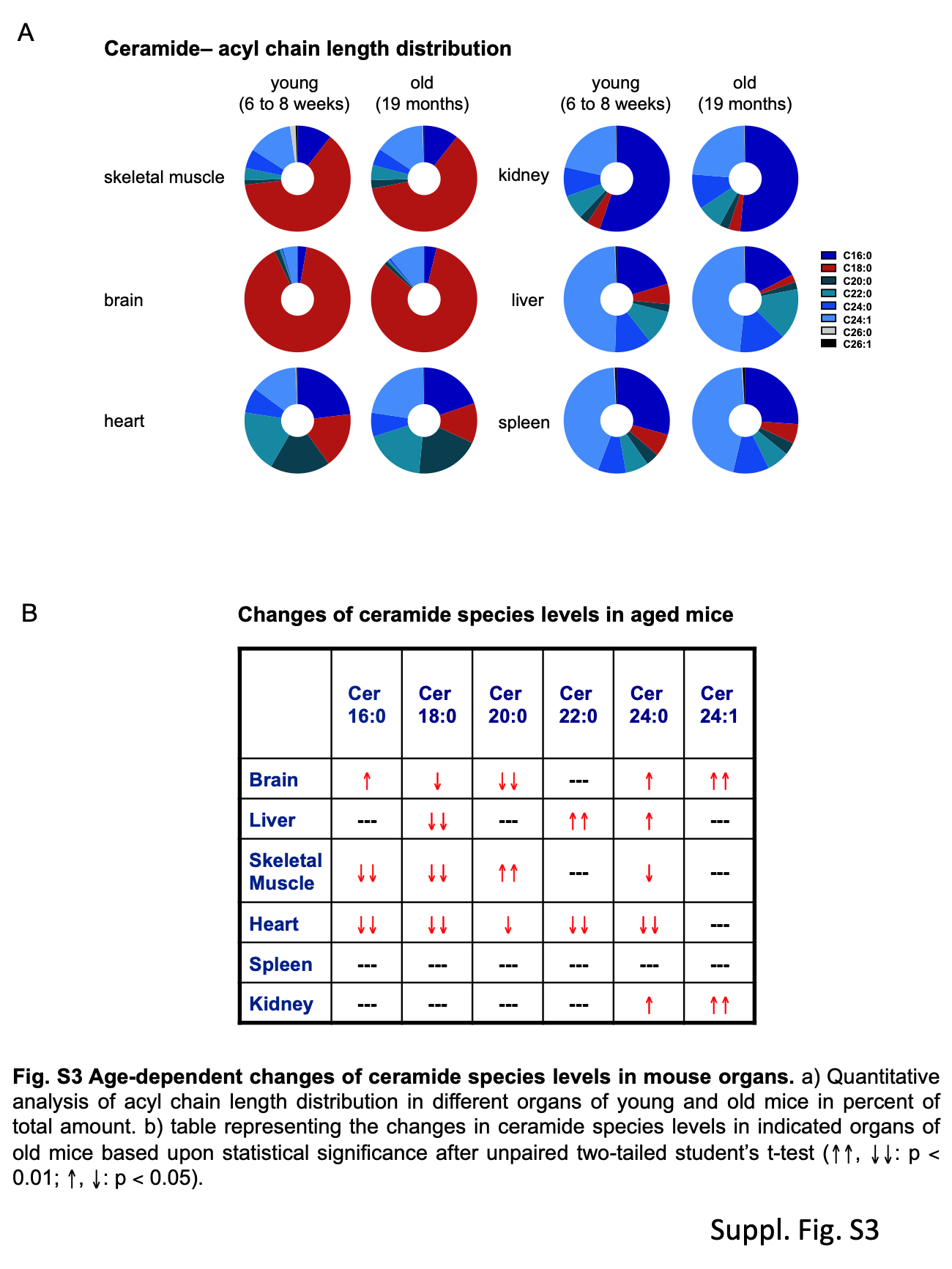

Supplement: Supplementary file 3 [file ACEL-19-e13049-s003.tiff]

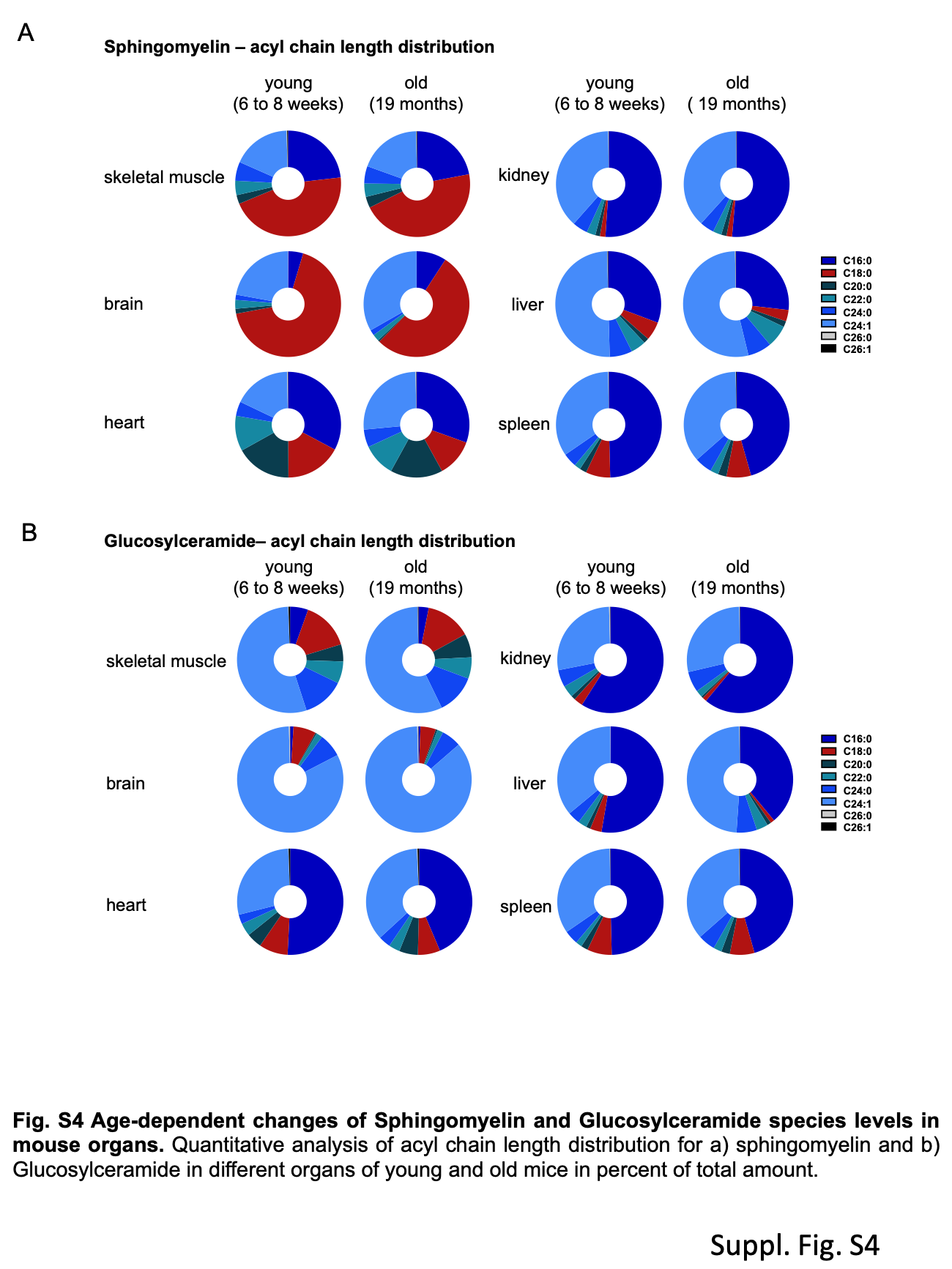

Supplement: Supplementary file 4 [file ACEL-19-e13049-s004.tiff]

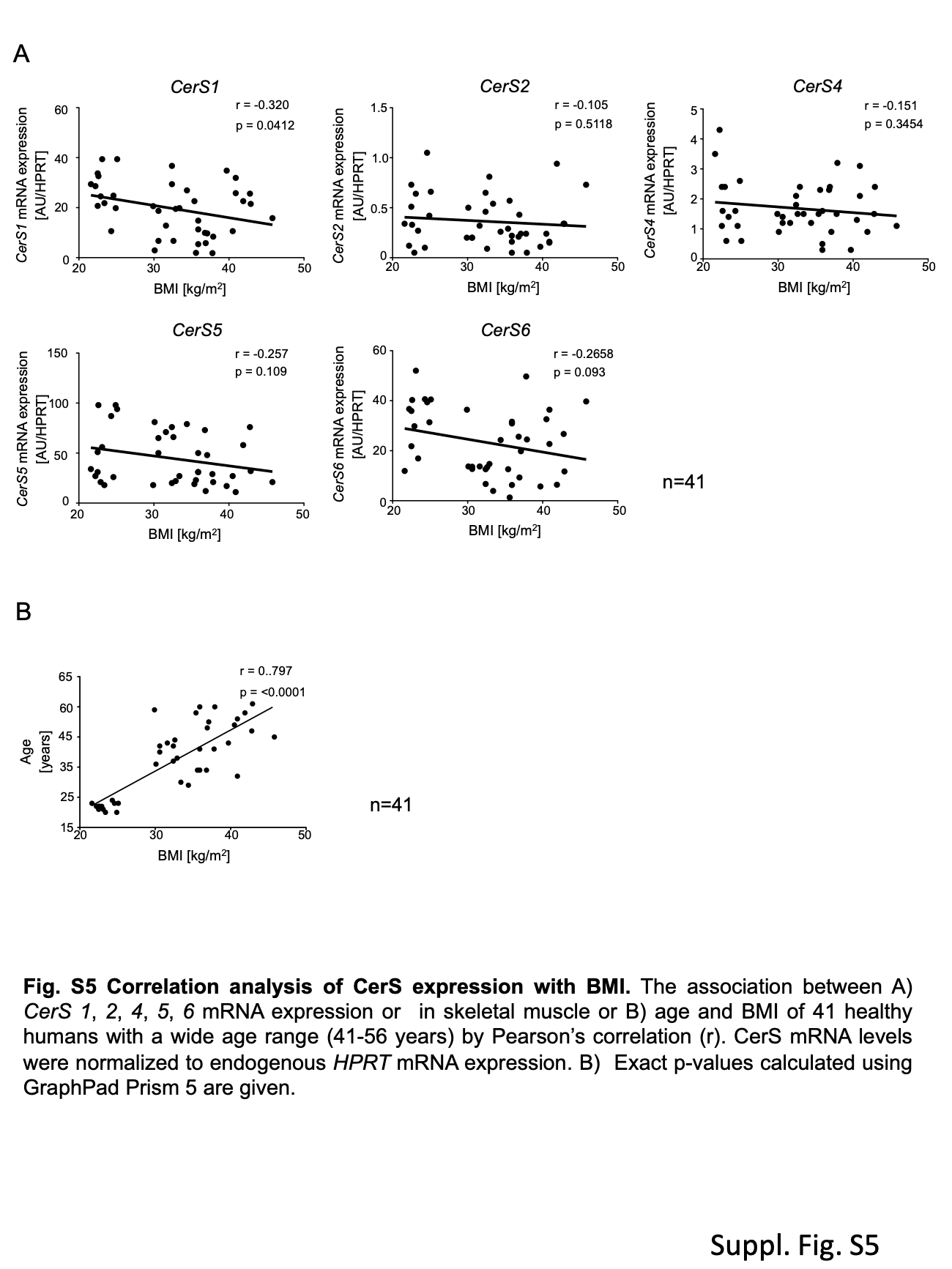

Supplement: Supplementary file 5 [file ACEL-19-e13049-s005.tiff]

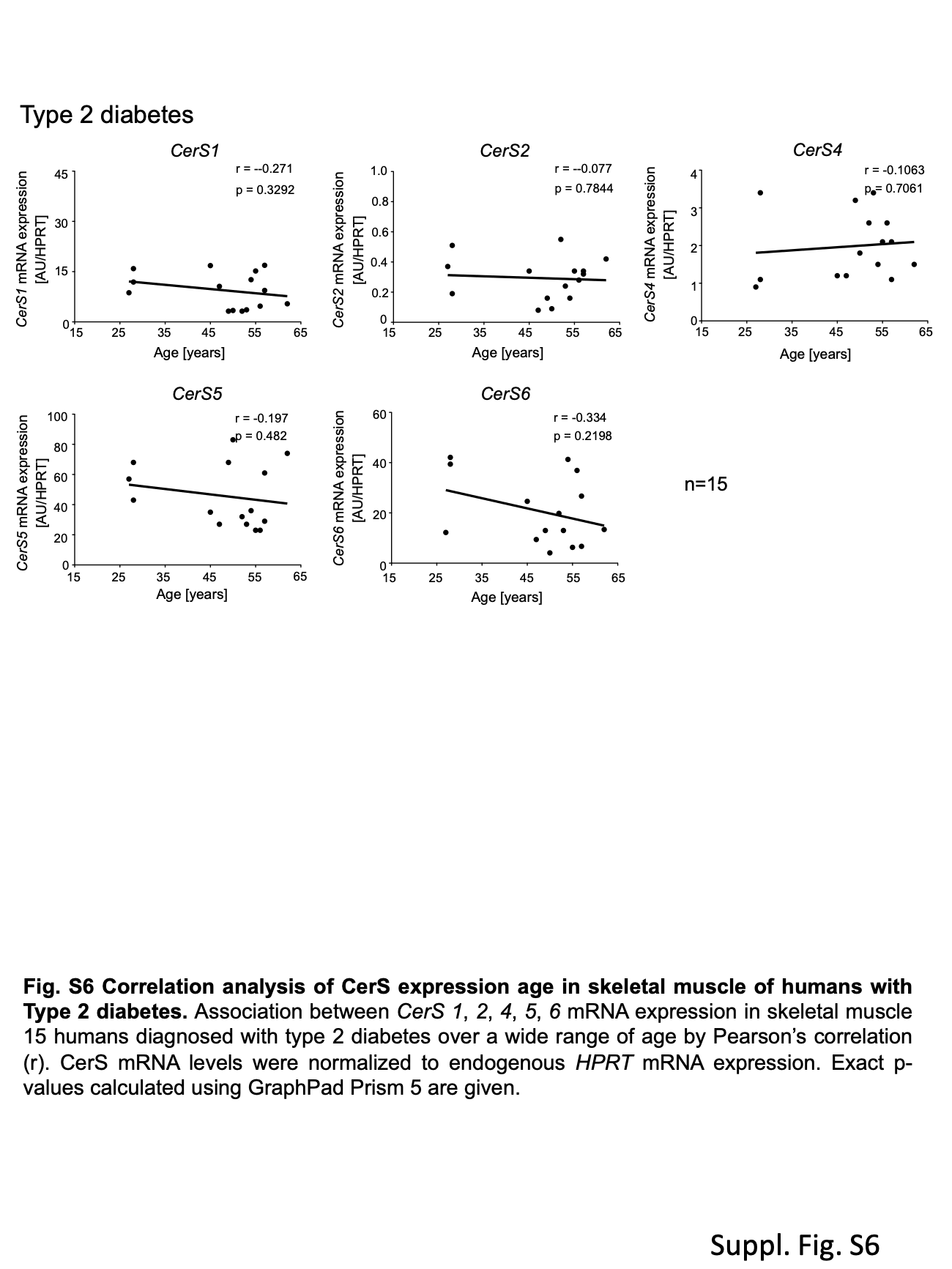

Supplement: Supplementary file 6 [file ACEL-19-e13049-s006.tiff]

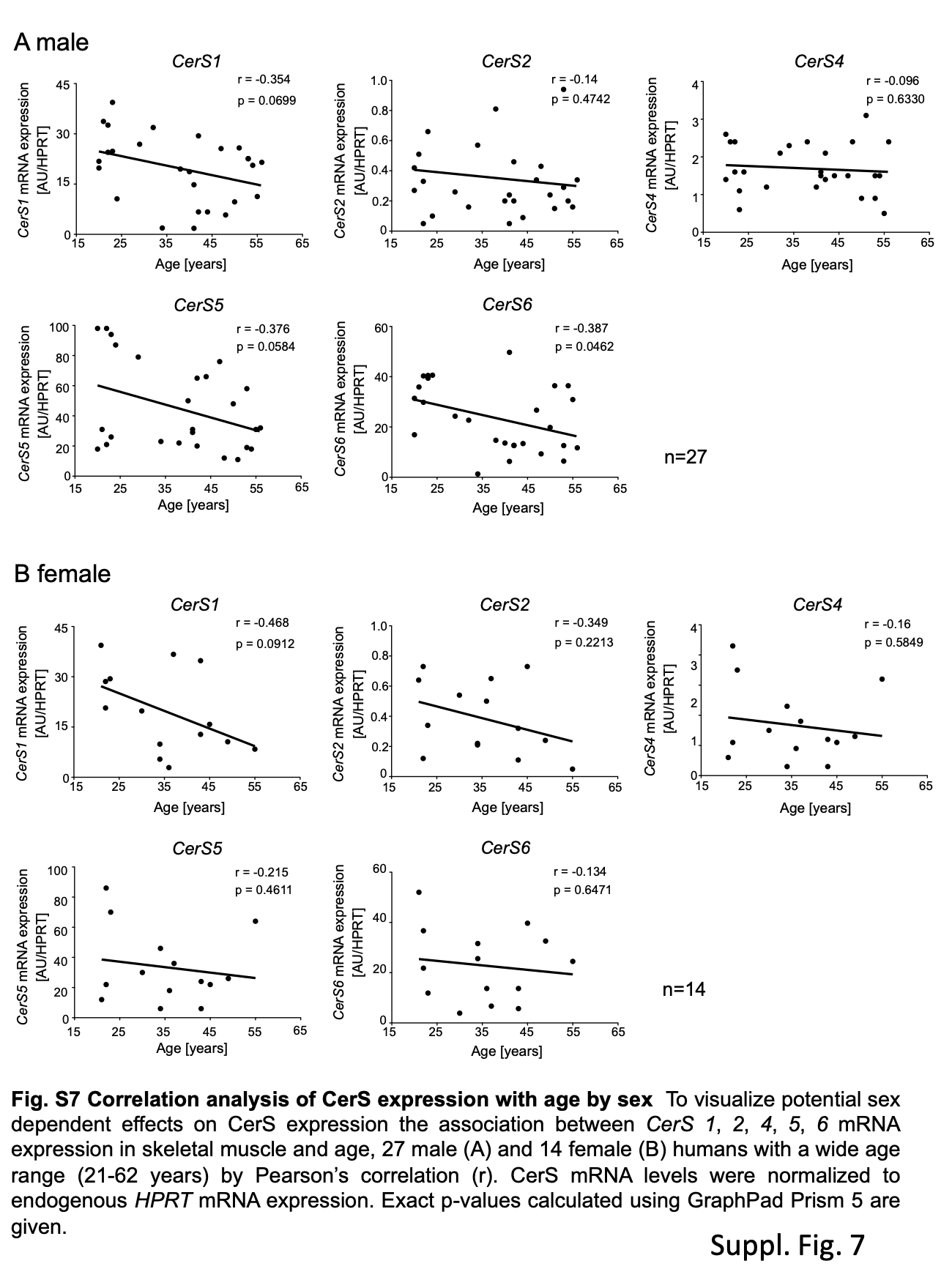

Supplement: Supplementary file 7 [file ACEL-19-e13049-s007.tiff]

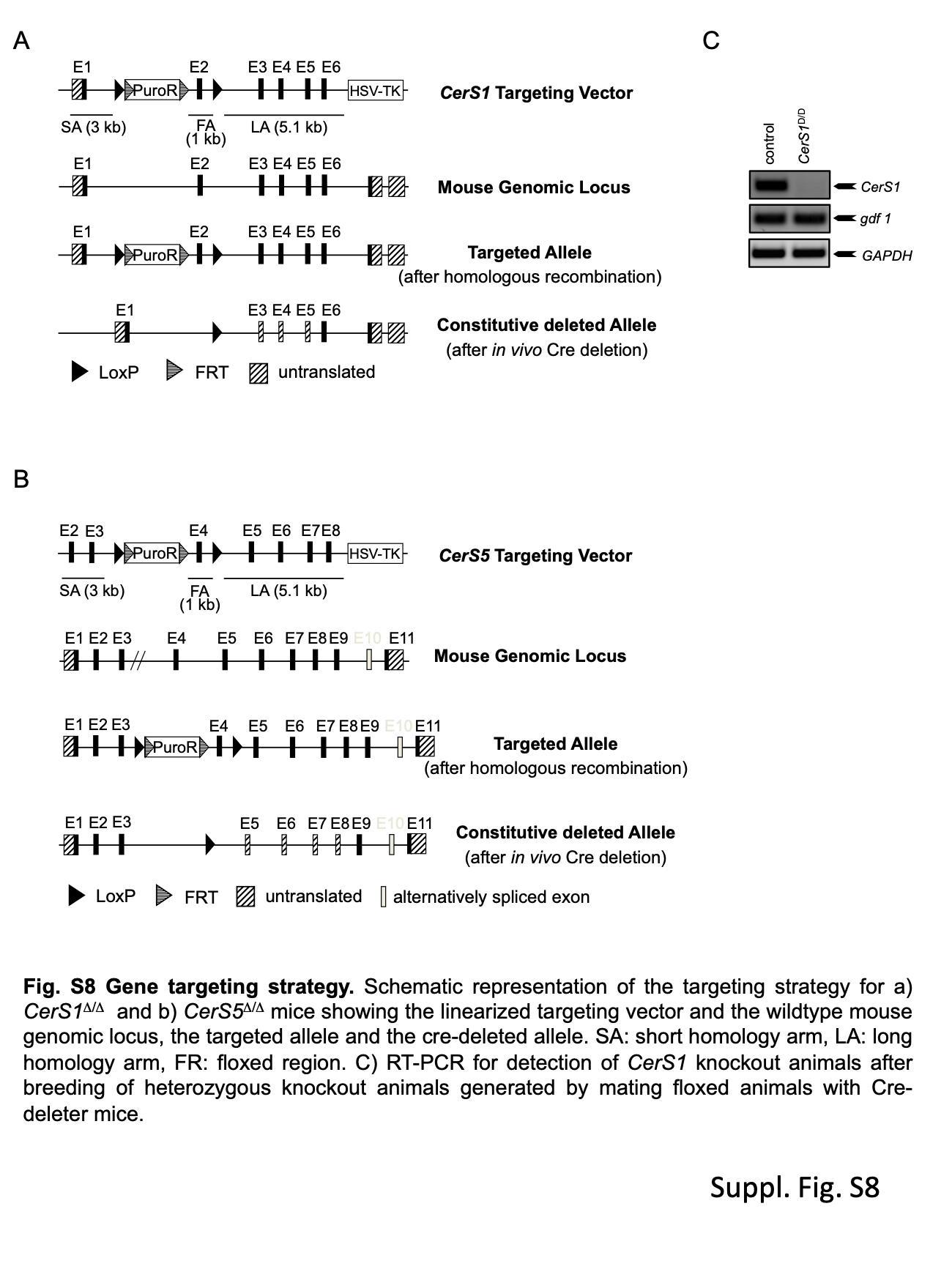

Supplement: Supplementary file 8 [file ACEL-19-e13049-s008.tiff]

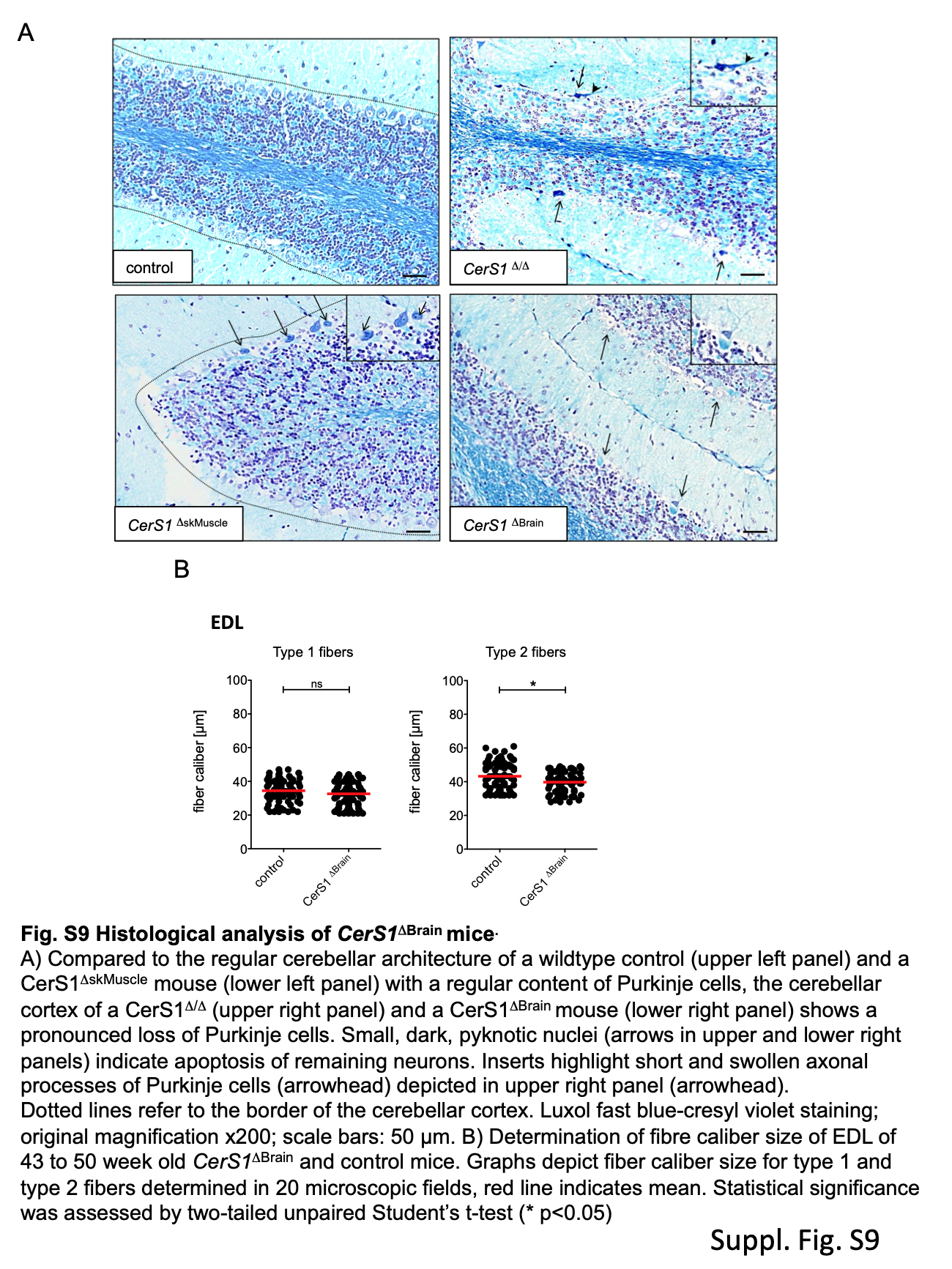

Supplement: Supplementary file 9 [file ACEL-19-e13049-s009.tiff]
