# Supplementary material for: A tissue‐specific screen of ceramide expression in aged mice identifies ceramide synthase‐1 and ceramide synthase‐5 as potential regulators of fiber size and strength in skeletal muscle
Source: Aging Cell. 2019 Nov 6;19(1):e13049. doi: 10.1111/acel.13049 (PMC6974707; doi:10.1111/acel.13049)
Supplement: Supplementary file 10 [file ACEL-19-e13049-s010.docx]

Supplemental Table S1

Characteristics of healthy human participants

| No. of participants | 41 |
| --- | --- |
| Age range | 20-56 |
| Age | 37±13^1^ |
| sex (m/f) | 27m / 14f |
| BMI (kg/m^2^) male | 32.5 ± 6.8^1^ |
| BMI (kg/m^2^) female | 32.4 ± 7.7^1^ |

^1^ values represent mean ± Standarddeviation
