# Supplementary material for: A tissue‐specific screen of ceramide expression in aged mice identifies ceramide synthase‐1 and ceramide synthase‐5 as potential regulators of fiber size and strength in skeletal muscle
Source: Aging Cell. 2019 Nov 6;19(1):e13049. doi: 10.1111/acel.13049 (PMC6974707; doi:10.1111/acel.13049)
Supplement: Supplementary file 11 [file ACEL-19-e13049-s011.docx]

Supplemental Table S2:

Characteristics of type 2 diabetes participants

| No. of participants | 15 |
| --- | --- |
| Age range | 27-62 |
| Age | 48 ± 11.4^1^ |
| sex (m/f) | 6m / 9f |
| BMI (kg/m^2^) male | 34 ± 5.0^1^ |
| BMI (kg/m^2^) female | 34 ± 4.1^1^ |

^1^ values represent mean ± Standarddeviation
