# Supplementary material for: A tissue‐specific screen of ceramide expression in aged mice identifies ceramide synthase‐1 and ceramide synthase‐5 as potential regulators of fiber size and strength in skeletal muscle
Source: Aging Cell. 2019 Nov 6;19(1):e13049. doi: 10.1111/acel.13049 (PMC6974707; doi:10.1111/acel.13049)
Supplement: Supplementary file 12 [file ACEL-19-e13049-s012.docx]

Supplemental Table S3:

Characteristics of patients with chronic heart failure

| age (years) | 69.1 ± 12.5^1^ |
| --- | --- |
| sex (m/f) | (5/1) |
| BMI (kg/m2) | 24.0 ± 2.2^1^ |
| Albumin (mg/dl) | 37.6 ± 2.2^1^ |
| Uric acid (mg/dl) | 6.0 ± 0.6^1^ |
| HB (g/dl) | 13.6 ± 1.3^1^ |
| CRP (mg/dl) | 1.6 ± 0.9^1^ |
| peak VO2 (ml/min/mg) | 23.2 ± 4.7^1^ |

^1^ mean ± standard deviation
