# Supplementary material for: A tissue‐specific screen of ceramide expression in aged mice identifies ceramide synthase‐1 and ceramide synthase‐5 as potential regulators of fiber size and strength in skeletal muscle
Source: Aging Cell. 2019 Nov 6;19(1):e13049. doi: 10.1111/acel.13049 (PMC6974707; doi:10.1111/acel.13049)
Supplement: Supplementary file 13 [file ACEL-19-e13049-s013.docx]

Supplemental Table S4

Characteristics of healthy volunteers for CHF control

| age (years) | 66.8 ± 21.1^1^ |
| --- | --- |
| sex (m/f) | (2/2) |

^1^ mean ± standard deviation
