# Supplementary material for: A tissue‐specific screen of ceramide expression in aged mice identifies ceramide synthase‐1 and ceramide synthase‐5 as potential regulators of fiber size and strength in skeletal muscle
Source: Aging Cell. 2019 Nov 6;19(1):e13049. doi: 10.1111/acel.13049 (PMC6974707; doi:10.1111/acel.13049)
Supplement: Supplementary file 14 [file ACEL-19-e13049-s014.docx]

Supplemental Table S5

Taqman assay No. and qRT-PCR primer sequences

| **Target** | **ABI Taqman Assay** |
| --- | --- |
| CerS1 | Hs04195319_s1 |
| CerS2 | Hs00371958_g1 |
| CerS4 | Hs00226114_m1 |
| CerS5 | Hs00908759_m1 |
| CerS6 | Hs00826756_m1 |
| HPRT1 | Hs01003267_m1 |
|  |  |
| **Target** | **Sequence 5' to 3'** |
| hGAPDH-for | AGCAATGCCTCCTGCACCAC |
| hGAPDH-rev | GTGGCAGTGATGGCATGGAC |
| hCerS1-for | ACCGACTACCCCTTCTTCCA |
| hCerS1-rev | GCGTAGATGGAGTGGCCATAG |
| hCerS2-for | GCCAGGTAGAGCGTTGGTTC |
| hCerS2-rev | GGCAATGAAGGCAATCAGGT |
| hCerS4-for | GCAGTATCAGCAAGTGTGCG |
| hCerS4-rev | CCTGTTGCTGATGGACTCGT |
| hCerS5-for | TGACACCCTTTTTGTGATCTTCA |
| hCerS5-rev | GAAGCATAAGGCCCGATTAT |
| hCerS6-for | GGGCGGACCTGAAGAACAC |
| hCerS6-rev | CGCACGGTTTGGCTACAAAT |
| mGAPDH-for | TCACCACCATGGAGAAGGC |
| mGAPDH-rev | GCTAAGCAGTTGGTGGTGCA |
| mGdf1-for | GGATGGCTGAAGTTCAGG |
| mGdf1-rev | ACAGCCAACCCCTGCAAA |
| CerS4-for | Peters, Vorhagen et al. 2015 |
| CerS4-rev | Peters, Vorhagen et al. 2015 |
| CerS1-for | Schiffmann, Birod et al. 2013 |
| CerS1-rev | Schiffmann, Birod et al. 2013 |
| CerS2-for | Schiffmann, Birod et al. 2013 |
| CerS2-rev | Schiffmann, Birod et al. 2013 |
| CerS6-for | Schiffmann, Birod et al. 2013 |
| CerS6-rev | Schiffmann, Birod et al. 2013 |
| CerS3-for | Schiffmann, Birod et al. 2013 |
| CerS3-rev | Schiffmann, Birod et al. 2013 |
| CerS4-for | Schiffmann, Birod et al. 2013 |
| CerS4-rev | Schiffmann, Birod et al. 2013 |
| CerS5-for | Schiffmann, Birod et al. 2013 |
| CerS5-rev | Schiffmann, Birod et al. 2013 |

Peters, F., S. Vorhagen, S. Brodesser, K. Jakobshagen, J. C. Bruning, C. M. Niessen and M. Kronke (2015). "Ceramide synthase 4 regulates stem cell homeostasis and hair follicle cycling." J Invest Dermatol **135**(6): 1501-1509.

Schiffmann, S., K. Birod, J. Mannich, M. Eberle, M. S. Wegner, R. Wanger, D. Hartmann, N. Ferreiros, G. Geisslinger and S. Grosch (2013). "Ceramide metabolism in mouse tissue." Int J Biochem Cell Biol **45**(8): 1886-1894.
